# Specialty management differences of syphilis and toxoplasmosis surrounding pregnancy: a prospective cross-sectional study

**DOI:** 10.1186/s12348-018-0152-9

**Published:** 2018-07-03

**Authors:** Jared S. Fredrickson, Jennifer Holmes, Jennifer N. Cathcart, Anne M. Lynch, Jason R. Kolfenbach, Alan G. Palestine

**Affiliations:** 1 Department of Ophthalmology, University of Colorado School of Medicine, 1675 Aurora Court, Mail Stop F731, Aurora, CO 80045 USA; 2 Department of Obstetrics and Gynecology, University of Colorado School of Medicine, 1635 Aurora Court, 3rd Floor, Aurora, CO 80045 USA; 3 Division of Rheumatology, Department of Medicine, University of Colorado School of Medicine, 1775 Aurora Court, Mail Stop B115, Aurora, CO 80045 USA

**Keywords:** Uveitis, Syphilis, Toxoplasmosis, Pregnancy, Management

## Abstract

**Background:**

Syphilis and toxoplasmosis are two infectious conditions that can occur during pregnancy. Both these diseases can have ocular manifestations and thus are treated by ophthalmologists and obstetricians. We hypothesized that specialty training would affect the way physicians selected therapy.

**Results:**

A total of 209 uveitis specialists and approximately 2500 obstetricians across the USA were surveyed using an online questionnaire distributed via listserv and social media posts. Survey respondents were given a series of clinical vignettes containing case examples of a female patient who was either contemplating pregnancy or in the first trimester and was diagnosed with either syphilis or toxoplasmosis. The questionnaire included a total of four case scenarios with questions relating to the management of these diseases, as well as pregnancy counseling. For the syphilis vignette, a total of 97 physicians responded to the survey questions. Choices of therapy between physician specialty differed significantly (*p* = 0.0001); however, pregnancy status did not seem to affect therapy choice in syphilis. A total of 96 physicians responded to the survey questions pertaining to the toxoplasmosis vignette. For a non-pregnant patient diagnosed with toxoplasmosis, the differences in therapy choice between specialties were not significant; however, when the patient was pregnant, therapy choice was significantly different between specialties (*p* = 0.0001).

**Conclusions:**

Differences exist between ophthalmologists and obstetricians concerning the therapy for syphilis and toxoplasmosis during pregnancy. Inter-specialty collaboration is needed to develop consistent criteria to improve the management of these patients.

## Background

Syphilis and toxoplasmosis are two serious infectious diseases which can occur during pregnancy. Both diseases have characteristic ocular findings that may be the only sign of active infection. Syphilis is the most common congenital infection worldwide, with approximately 1 million cases annually. Its prevalence is increasing in the USA, currently about 8–12/10,000 live births [1–3]. Likewise, the estimated worldwide prevalence of congenital toxoplasmosis is 1–4 per 10,000 live births [4, 5]. Both of these diseases can have equally devastating effects on fetuses and should be recognized and treated early during pregnancy.

Diagnosis can be made by an obstetrician or an ophthalmologist. It is known, however, that many conditions are managed differently by varying specialties [6–11]. We hypothesized that obstetricians and ophthalmologists would manage pregnant patients with syphilis and toxoplasmosis differently from one another. In order to investigate this hypothesis, we created an online survey to assess the management strategies of these differing specialties.

## Methods

We developed an online questionnaire that included case examples of a patient with ocular syphilis or toxoplasmosis. Each scenario included a female patient diagnosed with either syphilis or toxoplasmosis who was contemplating pregnancy or was currently in the first trimester of pregnancy. The questionnaire included a total of four case scenarios with questions relating to the management of these diseases, as well as counseling concerning pregnancy.

We used Research Electronic Data Capture (REDCap) to distribute the questionnaire to 209 members of the American Uveitis Society listserv and to approximately 2500 obstetricians via social media groups with a clickable link to the survey. All data were collected voluntarily and anonymously. The survey respondents were asked to identify whether they were an ophthalmologist or an obstetrician. They were then given the following clinical vignette: You are consulted about a 26-year-old female patient with newly diagnosed uveitis with a positive syphilis IgG, an RPR of 1:512, and a negative CSF RPR who wishes to get pregnant in the next year. Vision is 20/50 OU with placoid syphilitic lesions in the posterior pole. On genital examination, she has raised gray lesions consistent with condyloma lata. She is HIV negative.

The respondents were then asked to select the best choice for treatment. Choices included intravenous (IV) penicillin for 3 weeks, intramuscular (IM) benzathine penicillin for 3 weekly doses, or oral penicillin. The respondents were then asked if they would suggest deferring pregnancy. The same clinical scenario was presented again, but the patient was 7 weeks pregnant. The respondents were asked again what, in their opinion, is the best choice for treatment and would they suggest terminating the pregnancy.

The following toxoplasmosis vignette was presented: You are consulted about a 26-year-old female patient with newly diagnosed toxoplasmosis retinitis one optic disc diameter away from the fovea in one eye who wishes to get pregnant in the next year. Vision is 20/50 in that eye with a one disc diameter lesion in the posterior pole. Toxoplasmosis IgM is positive and IgG is negative. She is HIV negative.

The respondents were then asked in their opinion, what is the best choice for treatment. Choices included oral sulfamethoxazole/trimethoprim (SMX/TMP) + pyrimethamine for 6–8 weeks, oral SMX/TMP + clindamycin for 6–8 weeks, oral SMX/TMP + pyrimethamine + prednisone + folinic acid for 6–8 weeks, oral SMX/TMP + clindamycin + prednisone for 6–8 weeks, oral azithromycin for 6–8 weeks, oral azithromycin + prednisone for 6–8 weeks, 20 mg prednisone for 6–8 weeks, or intravitreal clindamycin and dexamethasone weekly for 1–3 times. The respondents were then asked if they would suggest deferring pregnancy. The same vignette was presented again, but the patient was 7 weeks pregnant and the respondents were asked what is the best choice for treatment and would they suggest terminating the pregnancy.

All data were analyzed using Statistical Analysis System (Cary, NC) version 9.4. The syphilis data were grouped into two groups: intravenous penicillin vs intramuscular penicillin, and chi-square analysis was performed on the resultant two by two tables. The toxoplasmosis data were grouped into local treatments (intravitreal injections) and oral treatments. The data were then analyzed using Fisher’s exact test. The toxoplasmosis data were further analyzed for pregnant vs non-pregnant treatment and controlled for by physician type. McNemar’s test was applied to the physician-controlled data to assess for statistical significance.

## Results

For the syphilis vignette, a total of 97 physicians responded to the survey questions, 57/209 (27% response rate) ophthalmologists and 40/2500 (approximately 1.5%) obstetricians. The choices between physician specialty differed significantly (*p* = 0.0001). As shown in Table 1, 80.7% of the ophthalmologists surveyed would treat the patient with IV penicillin and 19.3% would treat with IM benzathine penicillin. On the other hand, only 15% of obstetricians would treat the patient with IV penicillin and 85% would treat the patient with IM benzathine penicillin.

**Table 1:** Comparison of treatment choices of syphilis in non-pregnant and pregnant patients

	Syphilis non-pregnant treatment^a^		Syphilis pregnant treatment^b^	
	Treatment type		Treatment type	
	IV penicillin	IM benzathine penicillin	IV penicillin	IM benzathine penicillin
Ophthalmologists *N* = 57	46 (80.7%)	11 (19.3%)	47 (82.5%)	10 (17.5%)
Obstetricians *N* = 40	6 (15.0%)	34 (85.0%)	9 (22.5%)	31 (77.5%)

^a^Chi-square: *χ*^2^ = 40.799 (*N* = 97), *p* = 0.0001

^b^Chi-square:*χ*^2^ = 34.626 (*N* = 97), *p* = 0.0001

When asked about deferring the pregnancy, 74.1% of ophthalmologists recommended deferring the pregnancy while 96% of obstetricians recommended differing the pregnancy.

The treatment choices between specialists when the patient with syphilis was pregnant were similar to the responses seen in the non-pregnant scenario, as 82.5% of ophthalmologists chose IV penicillin and 17.5% chose IM benzathine penicillin. In pregnancy, 22.5% of obstetricians chose IV penicillin and 77.5% chose IM benzathine penicillin (*p* = 0.0001). Only 2% of ophthalmologists and 7% of obstetricians changed from IM penicillin to IV penicillin when treating a pregnant patient. When asked about terminating the pregnancy, 36% of ophthalmologists and 52% of obstetricians stated they would recommend terminating the pregnancy. 1.7% of ophthalmologists declined to answer.

For the toxoplasmosis vignette, a total of 96 physicians responded to the survey questions, 58 ophthalmologists (28% response rate) and 38 obstetricians (approximately 1.5% response rate). As shown in Table 2, 10.3% of the ophthalmologists surveyed would choose to treat a non-pregnant patient with local therapy (intravitreal injections) while 89.7% would treat with an oral agent. All but one of the obstetricians surveyed (97.4%) indicated that they would treat with an oral agent (*p* = 0.238).

**Table 2:** Comparison of treatment choices of toxoplasmosis in non-pregnant and pregnant patients

	Toxoplasmosis non-pregnant treatment^a^		Toxoplasmosis pregnant treatment^b^	
	Treatment type		Treatment type	
	Intravitreal treatment	Oral agent	Intravitreal treatment	Oral agent
Ophthalmologist *N* = 58	6 (10.3%)	52 (89.7%)	41 (70.7%)	17 (29.3%)
Obstetricians *N* = 38	1 (2.6%)	37 (97.4%)	1 (2.6%)	37 (97.4%)

^a^Fisher’s exact: probability (*P*) = 0.129 (*N* = 96), *p* = 0.238

^b^Chi-square: *χ*^2^ = 43.211 (*N* = 96), *p* = 0.0001

When asked about deferring pregnancy, 60% of ophthalmologists and 94% of obstetricians recommended deferring pregnancy.

When asked about the treatment of toxoplasmosis in pregnancy, 70.7% of ophthalmologists would treat with intravitreal injections and 29.3% would choose an oral agent. Of the obstetricians surveyed, 97.4% would treat with an oral agent and 2.6% (one physician) would recommend intravitreal injections (*p* = 0.0001). Table 3 shows that ophthalmologists, but not obstetricians, significantly changed their choice of treatment based on pregnancy status in that 63.8% of ophthalmologists changed their choice of therapy from oral to intravitreal injections whereas only 2.6% of obstetricians made a similar change. When asked about terminating the pregnancy, 24% of ophthalmologists and 57% of obstetricians recommended terminating the pregnancy.

**Table 3:** Change in treatment decision for toxoplasmosis based on pregnancy status

		Ophthalmologists^a^ *N* = 58		Obstetricians^b^ *N* = 38	
		Pregnant		Pregnant	
		Intravitreal treatment	Oral agent	Intravitreal treatment	Oral agent
Non-pregnant	Intravitreal treatment	4 (6.9%)	**2 (3.5%)**	0 (0.0%)	**1 (2.6%)**
	Oral agent	**37 (63.8%)**	15 (33.3%)	**1 (2.6%)**	36 (94.7%)

Bold type indicates the percent of physicians choosing to change therapy depending on the pregnancy status of the patient

^a^McNemar’s test statistic (S) 31.4103 *p* = 0.0001

^b^McNemar’s test statistic (S) 0.0000 *p* = 1.0000

## Discussion

While syphilis and toxoplasmosis have a well-established history for treatment options, the results of this study clearly demonstrate that there is a specialty-dependent difference between ophthalmologists and obstetricians in the management of these diseases in pregnant and non-pregnant patients. It is also clear that there is a difference in the perception of appropriate treatment based on specialty training and practice.

In the syphilis vignette, the patient presented with uveitis as well as other findings of syphilis. Whether the presence of uveitis in syphilis should be regarded as neurosyphilis is unclear based on the existing literature. Many argue that any ocular involvement in syphilis should be regarded as neurosyphilis [12–15], while others argue that only posterior uveitis involving tissue derived from neuroepithelium (optic nerve, retina) should be considered neurosyphilis [12]. In 2016, CDC announced a clinical advisory stating that ocular syphilis should be treated according to the treatment recommendations for neurosyphilis [16]. The distinction is important because neurosyphilis should be treated with IV aqueous crystalline penicillin 18–24 million units per day, for 10–14 days, whereas other forms of syphilis can be adequately treated with IM benzathine penicillin G [17]. It has been shown that Treponemal pallidum organisms can be sequestered in the CNS and aqueous humor and that certain formulations of penicillin do not maintain adequate drug levels in these sites [17, 18].

In the syphilis vignette, the patient was presented as having a syphilitic lesion in the posterior pole which most ophthalmologists consider at least a serious vision-threatening condition and possible indication of neurosyphilis as evidenced by a large majority of ophthalmologists opting to treat the patient with IV penicillin in our study. In contrast, most obstetricians chose to treat the non-pregnant patient with IM benzathine penicillin rather than IV penicillin. Obstetricians may be less familiar with these recommendations to treat syphilis with ocular involvement as neurosyphilis. This topic is discussed in ophthalmologic journals but likely gets little or no attention in obstetrical literature. Ocular inflammation may be the only clinical sign in a patient with early neurosyphilis, and CNS involvement may range from 16 to 60%. There is some debate on the criteria for CSF testing in ocular disease, and no single CSF test will reliably exclude neurosyphilis in all patients [19]. Hence, it is reasonable to approach all ocular syphilis, especially if the posterior segment is involved, as possible neurosyphilis. These management differences have the potential to impact patient care, not only the mother but also the future fetus [20]. However, the financial, logistical, and time burdens of the recommended IV infusions for 2–3 weeks to treat possible neurosyphilis are significant when compared to 3 weekly IM injections.

Pregnancy did not seem to affect either specialty’s management decisions concerning syphilis as a small percentage of physicians changed from IM penicillin to IV penicillin. It has been shown that the recommended treatment regimens are effective in maternal and fetal disease [3, 21].

While pregnancy did not seem to affect the choice of management in syphilis, it greatly affected the choices ophthalmologists made in managing toxoplasmosis. For a non-pregnant patient, a large majority of ophthalmologists selected an oral therapy, but when the same patient was pregnant, less than a third of ophthalmologists recommended an oral agent. The presence of a fetus did not affect obstetricians management strategy as all but one obstetrician recommended systemic agents whether the patient was pregnant or not. This difference in management has important potential implications. First, by only selecting local therapy (intravitreal injections) to treat toxoplasmosis in pregnancy, this strategy does not address the possibility of systemic infection and offers little protection to the fetus since the patient was IgM positive indicating recently acquired toxoplasmosis. Second, it highlights the clear differing practice patterns that these specialties have of prescribing systemic medications in pregnancy. Our data does not explain why most ophthalmologists opted to switch to intravitreal injections in pregnancy, but it is likely that there was concern regarding harm to the fetus with potential side effects of systemic treatments.

One limitation to our study was our small sample size and low response rate. This could have introduced bias into our study although the differences are highly significant. As most general obstetricians rarely treat these conditions, the responses may not reflect the complete obstetrical opinion, as there was only a small sampling of physicians. Our survey was also largely distributed to physicians in academia, and this may make our results less generalizable to those outside of academic medicine.

## Conclusion

The results of this study clearly demonstrate that there are differences between ophthalmologists and obstetricians when it comes to managing syphilis and toxoplasmosis during pregnancy. It is also clear that the two groups have differing practice patterns when selecting systemic treatments for these diseases during pregnancy. It may be worthwhile for specialty societies to collaborate on developing consistent criteria to improve the management of these patients.
